# TDNN achitecture with efficient channel attention and improved residual blocks for accurate speaker recognition

**DOI:** 10.1038/s41598-025-09386-0

**Published:** 2025-07-02

**Authors:** Wenzao Li, Sai Yao, Bing Wan, Linsong Xiao, Chengyu Hou, Yanchuan Zhong, Wengang Zhou

**Affiliations:** 1 School of Communication Engineering, Chengdu University of Information Technology, Chengdu, 610225 Sichuan China; 2 School of Software, Chengdu Polytechnic, Chengdu, 610225 China; 3 Sichuan Provincial Climate Center, Chengdu, 610072 China; 4 School of Flight Technology, Civil Aviation Flight University of China, 46 Nanchang road, Guanghan, 618307 China

**Keywords:** Electrical and electronic engineering, Computer science

## Abstract

In recent years, with the advancement of deep learning, Convolutional Neural Networks (CNNs) have been widely applied in speaker recognition, making CNN-based speaker embedding learning the predominant method for speaker verification. Time Delay Neural Networks (TDNN) have achieved notable progress in speaker embedding tasks. However, TDNN often struggles with accurately modeling multi-scale features when processing complex audio data, which can result in reduced speaker recognition accuracy. To address this issue, we propose the Efficient Parallel Channel Network - Time Delay Neural Network (EPCNet-TDNN), building upon the ECAPA-TDNN architecture. The proposed model incorporates a novel Efficient Channel and Spatial Attention Mechanism (ECAM) in the ECA_block, which replaces the original SE_block. This modification enhances the model’s ability to capture key information, improving overall performance. To further reduce feature dependency and enhance multi-scale information fusion, a Parallel Residual Structure (PRS) is introduced, enabling the independent capture of multi-scale features through parallel computation instead of sequential processing. The ECA_block adopts the output structure of ECAPA-TDNN, Calling it a Tandem Structure (TS). Facilitating the integration of information from different scales and channels, resulting in more refined feature representations. After multi-scale feature extraction, the Selective State Space (SSS) module is introduced to improve the model’s ability to capture temporal sequence features. Experimental results on the CN-Celeb1 dataset show that EPCNet-TDNN has a relative improvement of about 14.1% (0.025), 9.4% (0.075), and 6.6% in EER, minDCF, and ACC, respectively, compared to ECAPA-TDNN. These results demonstrate the significant improvements achieved by the proposed approach over previous methods.

## Introduction

Speaker recognition (SR) is a modern biometric technology that converts captured acoustic features into spectrograms^1^. These spectrograms are then compared with stored spectrograms to determine if they belong to the same identity, thus enabling identity verification. As a popular research direction in the field of speech processing, SR enables computers to accurately identify a speaker’s voice and analyze the acoustic information contained therein, further improving the accuracy of speech processing. The technology offers advantages such as non-contact operation, high convenience, high security, low cost, and support for remote authentication, making it widely used in fields like banking transaction security, remote payment authentication, criminal suspect identification, and automatic identity tagging. Traditionally, i-vector^2^ combined with probabilistic linear discriminant analysis (PLDA)^3^ has been a widely used method. With the booming development of deep learning, optimizing the accuracy and robustness of voiceprint recognition has become an important research direction, and researchers have made significant efforts to improve network backbones, pooling mechanisms, and loss functions^4^.

Convolutional Neural Networks (CNNs)^5^ are the current mainstream framework, with two main branches: Time Delay Neural Networks (TDNN)^6^ based on one-dimensional convolution (Conv1d), and ResNet^7^ based on two-dimensional convolution (Conv2d). TDNN can process variable-length sequences and capture both short-term and long-term dependencies in the temporal dimension. TDNN demonstrates strong temporal modelling capabilities in sequence data, such as speech, by applying the same convolution kernel at different time points. X-vector^8^ and its variants were proposed to further enhance performance. Subsequently, ECAPA-TDNN^9^ further improved speech feature extraction by introducing several architectural enhancements to the original X-vector, making it one of the most widely adopted models in tasks such as voiceprint recognition. Building upon ECAPA-TDNN, Han et al. introduced a multi-resolution feature encoder as an additional branch to improve performance in short speech segment conditions. Similarly, Liu et al. proposed an attention-based pre-segment module to enhance feature representation, which they named MFA-TDNN^10^. On the other hand, ResNet^7^, a Conv2d-based network architecture proposed by He et al. for image classification tasks, introduced residual modules that successfully address the gradient vanishing problem in deep networks. This allows deep networks to be trained more stably and has led to its widespread use in image processing and computer vision tasks^11^. ResNet has also been gradually introduced into speech processing due to its powerful feature extraction capabilities. Based on the ResNet model, Kynych, F et al. developed a real-time speaker recognition system using SE-ResNet-34 embedding and online clustering technology. This system achieves accuracy comparable to offline systems with low latency and low computational cost. It was subsequently extended to multimodal audio-visual processing for broadcast stream analysis^12^. However, these methods still have room for improvement in capturing temporal information, as well as in multi-scale feature extraction, especially in complex scenes. Therefore, it remains a valuable task to design a model that improves multi-scale feature extraction and enhances the ability to capture temporal information.

The two main challenges facing models with multi-scale feature extraction capability and enhanced ability to capture temporal information are how to efficiently capture key information in order to ensure the accuracy of the model, and how to maintain an efficient temporal modeling capability to avoid excessive computational complexity when dealing with complex temporal data. To address these challenges, the EPCNet-TDNN algorithm is proposed in this paper. The main contributions of this paper are as follows:

1. Propose the ECA_block module to replace the original SE_block: by introducing the novel ECAM mechanism in ECA_block to replace the original SE_block, and replacing the ordinary one-dimensional convolution in SE_block with a deep separable convolution, the computational efficiency and feature capture ability of the model are significantly improved, further enhancing the overall performance.
2. Parallel residual structure is designed: in order to reduce the dependence between features and enhance the fusion of multi-scale information, this paper proposes a parallel Res2Net structure, which enables the model to capture multi-scale features independently through parallel computing and improves the model’s multi-dimensional feature capture.
3. Drawing on the output structure of ECAPA-TDNN: The output structure design of ECAPA-TDNN is introduced in ECA_block, which improves the model’s ability to integrate different scales and channel information by mimicking the multi-level feature fusion and further enhances the feature representation.
4. Introduction of Selective State Space (SSS) module: after multi-scale feature extraction, this paper introduces the SSS module, by introducing the SSS module, it improves the model’s modelling of long and short-term dependencies, enhances the ability to understand complex time-series data, and significantly improves the performance in dynamic data. Finally, our proposed model is verified to be valid on the dataset Cn-Celeb1. Experimental results on the CN-Celeb1^13^ dataset show that EPCNet-TDNN has a relative improvement of about 14.1% (0.025), 9.4% (0.075), and 6.6% in EER, minDCF, and ACC, respectively, compared to ECAPA-TDNN.

## Related works

Voiceprint recognition is a key research direction in speech signal processing, aiming to accurately recognize and verify the identity of speakers using advanced algorithms. In recent years, researchers have done a great deal of work in acoustic pattern recognition. In terms of front-end acoustic feature extraction, most of the existing acoustic feature extraction methods are mainly based on some form of short-time spectra to achieve speaker verification for short speech, such as Mel Frequency Cepstrum Coefficients (MFCC)^14^, Linear Predictive Cepstrum Coefficients (LPCC)^15^, and other acoustic features. In addition, the Gaussian Mixture Model-Universal Background Model (GMM-UBM)^16^, based on statistical methods, is a traditional mainstream method for short speech acoustic recognition. Its main idea is to calculate the new parameters of the UBM model from the training data of the target speaker, and then adapt the newly obtained parameters to the original parameters of the UBM model to get the final target speaker model. Following this, Campbell et al. proposed a Gaussian Mixture Model Super Vector Support Vector Machine (GMM-GSV)^17^. Kenny et al. proposed a joint subspace alternative learning method called Joint Factor Analysis (JFA) and introduced the i-vector method. The core idea was to construct a low-dimensional channel-dependent speaker space by defining the total variability space^18^. Al-Kaltakchi et al. proposed a method based on the combination of i-vectors and extreme learning machines, which were used to improve the robustness of speaker recognition models^19^.

With the further development of deep learning, a variety of voiceprint recognition methods have been proposed. Povey et al. proposed a factorized decomposition time delay neural network (F-TDNN)^20^, which improves training efficiency by decomposing the parameter matrix of the TDNN into smaller matrices. In addition, Snyder et al. proposed an extended time delay neural network (E-TDNN)^21^, which is based on a wider and deeper structure that enables the model to learn more information and significantly improves the performance of speaker recognition. F. Daneshfar et al. proposed a speech emotion recognition system based on speech and glottal signals, extracting features using Gabor filter banks (GBFB and SGBFB) and employing a hierarchical adaptive weighted multi-layer extreme learning machine (H-AWELM) for classification, addressing the data imbalance issue in multi-class ELM training. The system was evaluated on the EMODB dataset, demonstrating excellent emotion recognition performance^22^. Li, Y et al. were inspired by human brain observations of noise speech spectrum maps and cognitive behaviour and proposed an auxiliary model speech enhancement framework that decomposes noise spectrum energy into regular and random components. Through the collaborative work of multiple sub-networks, such as voiceprint segmentation networks and noise reconstruction networks, they achieved state-of-the-art speech enhancement performance on public datasets^23^. Li, YF et al. proposed a DS-TDNN^24^ model, which extracts both local and global features in parallel by introducing a global perceptual filtering layer (GF layer) and using a dual-stream architecture. Combining dynamic filtering strategies and sparse regularization methods to enhance the model’s ability to capture long-time dependencies and reduce computational costs, better performance and efficiency are achieved in speaker verification tasks. Zhang, HJ et al. addressed the issue of accuracy degradation in voiceprint recognition across different scenarios and channels by proposing a deep learning-based dual-channel voiceprint recognition model. Through the design of the DWLoss loss function, ECA feature extraction module, and PLDA channel compensation technology, the model’s noise resistance and recognition accuracy were significantly improved^25^. In the loss function section, Ji, CQ, and others addressed issues such as training-testing inconsistency, sample imbalance, and similarity overlap in traditional Softmax for speaker verification. They proposed the SphereSpeaker adaptive objective function (introducing an angular margin mechanism) and the ResNet-P network architecture. Experimental results show that this method achieves the lowest equal error rate and significantly improves the performance of speaker verification systems^26^. Han et al. addressed the training-verification inconsistency in traditional classification-based speaker embedding by proposing score comparison-based learning, which enforces lower intra-class and higher inter-class variance at the similarity score level. They also introduced a generalized loss function unifying various conventional losses and regularization techniques, demonstrating improved performance and robustness against overfitting across multiple datasets^27^. In addition, Desplanques et al. proposed ECAPA-TDNN, a TDNN-based speaker verification model, which introduces aggregated information, channel attention, and improved propagation methods to further improve the robustness of the speaker recognition system^9^. Based on ECAPA-TDNN, Lin et al. proposed the ECAPDLA CNNv2-TDNN^28^ model, which significantly improves the feature extraction capability and training by introducing a pre-activated convolutional layer (CNN stem), changing the multilayer aggregation to Deep Layer Aggregation, and replacing the SE-Res2block with a self-calibrating block (SC block). Liu et al. proposed DF-ResNets and DF-ECAPAs models^4^, which significantly improve the model depth and performance by introducing depth-first (DF) architectural design rules. They also proposed two attentional feature fusion schemes (S-AFF and P-AFF), which significantly enhance the performance of smaller models with low computational cost, striking a balance between performance and complexity. Luo et al. extended the convolutional receptive field by introducing a multi-scale channel adaptive module (MSCA-Res2Block) and combined it with a balanced fine-tuning strategy and Z-Score normalization, which significantly improved the performance of Arabic dialect recognition^29^.

Despite the significant progress made by existing methods in voiceprint recognition, there are still some shortcomings. Many models have difficulty in adequately capturing multi-scale information during feature extraction, especially when dealing with more complex speech signals, and have limited ability to model long- and short-term dependencies. In addition, the models still need improvement in coping with the capture of global information and adapting to different speech features. Our experimental results on the Cnceleb1 dataset demonstrate the potential of the model to improve recognition accuracy and robustness, especially when dealing with complex scenes and short speech, showing good promise. However, further research and validation on more datasets will help to comprehensively evaluate the model’s generalization ability and performance in real-world applications.

## Proposed method

This section provides a detailed description of the improved EPCNet-TDNN, beginning with the widely used ECAPA-TDNN network architecture. Next, the evolution from ECAPA-TDNN to the enhanced EPCNet-TDNN is outlined. Finally, each innovation introduced in the improved model is presented step by step.

### ECAPA-TDNN

ECAPA-TDNN is an enhanced version of the x-vector architecture that has demonstrated outstanding performance in several speaker verification competitions, including VoxSRC-2019^30^, VoxSRC-2020^31^, SdSVC-2021^32^, and VoxSRC-2021^33^. ECAPA-TDNN employs a 1D convolutional layer, where the input feature is a 2D representation in the form of F$\times$T, with F representing the frequency dimension and T representing the time dimension. The input is first processed through a 1D inflated convolutional layer, producing a feature map of size C$\times$T, where C denotes the number of channels. This feature map is passed through three consecutive SE-Res2Blocks, each comprising two 1D inflated convolutional layers, a 1D inflated Res2Block, and a 1D squeeze-and-excitation (SE) block.

**Fig. 1:**
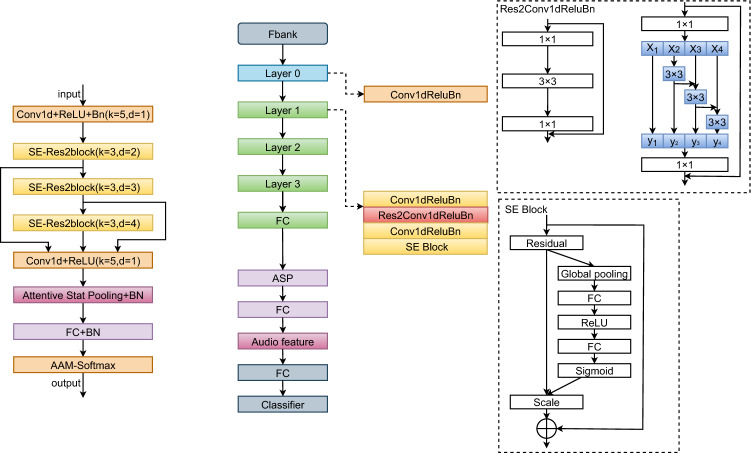
Structure of ECAPA-TDNN.

In addition, a multilayer feature aggregation module is specifically designed to capture and integrate the hierarchical speaker information from different network layers by linking the output feature maps of the three SE-Res2Blocks. Following this, an attention-based statistic pooling layer is applied to emphasize speaker-specific attributes across both the channel and temporal dimensions using a self-attention mechanism. Lastly, similar to ResNet-based speaker verification systems, a fully connected layer is used to reduce the dimensionality of the pooled vectors, and AAM-softmax serves as the loss function during model training, as illustrated in Fig. 1.

Although the ECAPA-TDNN model excels in audio feature extraction, it still exhibits limitations in capturing temporal information, enhancing the flexibility of the attention mechanism, and deepening feature fusion. The model primarily integrates multi-level feature information through basic feature concatenation and an attention mechanism. However, the fusion process for multi-scale information remains relatively simple, and the model’s capacity for feature selection is constrained, making it challenging to effectively handle multi-level information across different types of audio.

### Improved ECA_block

In this section we explain the improved ECA_block in detail, including the ECAM mentioned therein, the depth separable convolution, the parallel residual structure and finally the use of tandem structure in the ECA_block.

#### ECAM

The Convolutional Block Attention Module (CBAM)^34^ is a lightweight and efficient attention mechanism that sequentially applies channel and spatial attention to enhance feature representation. The channel attention module identifies “which features are more important” by generating channel-level attention weights through global average pooling and max pooling, while the spatial attention module highlights “which locations to focus on” by deriving spatial attention weights based on channel information. This two-step attention mechanism enables the model to concentrate on more informative features, thereby improving performance across various tasks with minimal computational overhead.

**Fig. 2:**
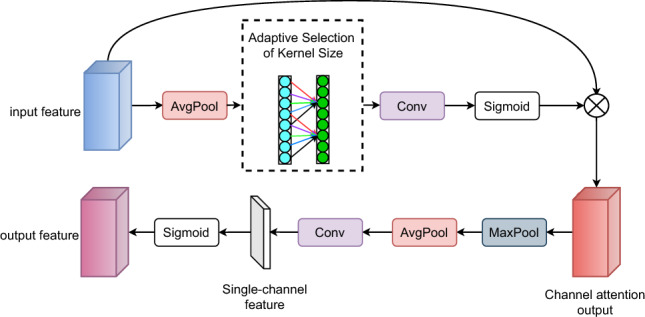
Structure of ECAM.

ECAM is designed by adopting the concept of CBAM, replacing the channel attention mechanism with Efficient Channel Attention (ECA)^35^ to optimize computational efficiency while preserving performance. ECA models inter-channel dependencies using local 1D convolution, eliminating the additional parameters and computational cost introduced by the fully connected layer in CBAM. This allows ECA to not only capture fine-grained channel relationships effectively but also mitigate information loss caused by global pooling. The structure of ECAM is illustrated in Fig. 2.

As illustrated in Fig. 2, the process begins with the input features being pooled by global averaging to capture global information, followed by a convolution operation via the Adaptive Selection of Kernel Size. The channel weights are generated by performing a rapid 1D convolution of size, where the size k is adaptively derived from a mapping of the channel dimension C. The module can automatically select convolution kernels of different sizes to capture features across different receptive field ranges and generate multi-scale feature representations^35^. Among them:

1 $$\begin{aligned} k = \varphi (C) = \text {make}\_\text {odd}\left( \left\lfloor \frac{|\log _2(C) + b|}{\gamma } \right\rfloor \right) \end{aligned}$$

where *C* = 512, $\gamma = 2$ and $b = 1$ , $\lfloor \cdot \rfloor$ denotes the floor function, $|\cdot |$ denotes the absolute value, and $\text {make\_odd}(\cdot )$ denotes the function that ensures the result is an odd number.

The function $\varphi$ adaptively determines the kernel size *k* through logarithmic mapping. It first calculates $\log _2(C) + 1$, then divides by $\gamma (= 2)$ and takes the floor, followed by a parity check to ensure *k* is an odd value. This design enables larger channel numbers to correspond to larger kernels, allowing the capture of longer-range inter-channel dependencies. The odd kernel size ensures the symmetry of convolution operations, which is beneficial for feature center alignment. When the number of channels is small, smaller kernels are used; as the number of channels increases, the kernel size grows accordingly, achieving adaptive cross-channel interaction modeling.

After the convolution operation, the features are passed through a Sigmoid activation function to generate the channel attention weights and are multiplied element-wise with the input features to complete the channel enhancement. Next, key information in the spatial dimension is captured, and spatial feature attention is then generated. Finally, channel attention and spatial attention are combined to produce the final channel attention result, enhancing the model’s ability to perceive key features and improving its selectivity for important information.

ECAM is used to replace the SE attention module in the original block, enabling the model to simultaneously focus on crucial channel and spatial features, thereby further enhancing its feature representation capability. Additionally, the design of ECAM improves the model’s accuracy and feature selectivity without increasing its complexity.

#### Depthwise separable convolution

Based on the traditional convolutional neural network, the deep separable convolution module (DSConv)^36^ effectively reduces the number of parameters and computational complexity by decomposing standard convolution into depthwise convolution and pointwise convolution. Specifically, depthwise convolution is first performed on each channel, followed by pointwise convolution to form the output feature map.

**Fig. 3:**
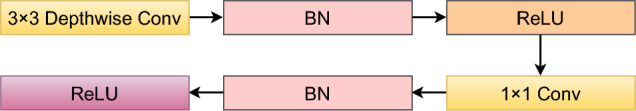
Structure of DSC.

As shown in Fig. 3. Spatial features are first extracted by a 3$\times$3 deep convolution operation, differentiating it from conventional convolution as it is performed independently for each input channel. After convolution, Batch Normalization (BN) is applied, which stabilizes the training process and improves the model’s generalization ability. Next, nonlinear properties are introduced through the ReLU activation function to enhance the expressive power of the model. Subsequently, 1$\times$1 convolution linearly combines information from different channels to achieve inter-channel interactions, further enhancing the model’s feature fusion capability while retaining computational efficiency. This process is followed once more by BN to ensure data distribution stability, and the ReLU activation function is re-applied to enhance the model’s nonlinear mapping ability.

In voiceprint recognition tasks, the speech signal encodes substantial time-frequency information, and the extraction of effective voiceprint features is crucial for accurately identifying individual IDs. The application of DSConv captures features across different frequency ranges with greater granularity and enables the model to process large-scale speech data more efficiently by reducing computational overhead. A key advantage of deep convolution is that it operates independently on each channel, thereby capturing unique patterns and preserving more detailed features.

#### Parallel residual structures

The Res2Net structure in ECAPA-TDNN enhances the model’s feature representation capability by processing input features through multi-scale groupings. Specifically, Res2Net divides the input features into multiple sub-feature groups, where each sub-group undergoes convolutional operations at its respective scale, and the results are fused through stepwise summation. This progressive feature fusion method effectively captures multi-scale information from local to global, improving the model’s ability to represent complex speech signals.

**Fig. 4:**
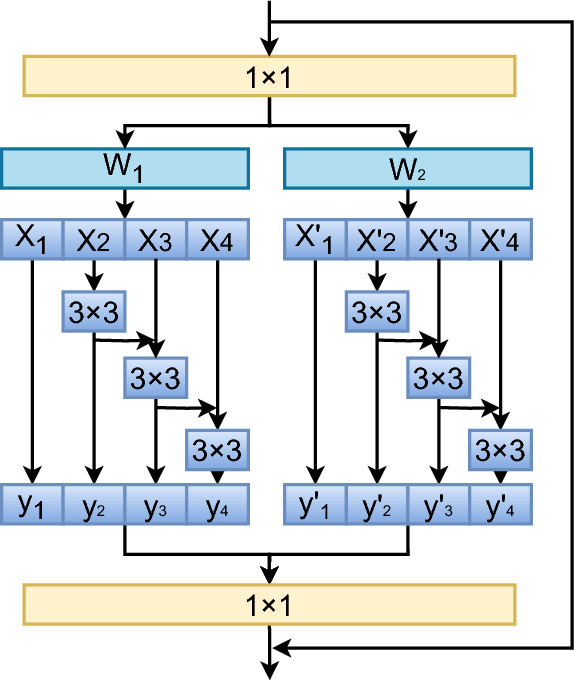
Structure of PRS.

In comparison to the Res2Net structure, the parallel residual structures proposed in this paper offers significant advantages in multi-scale feature fusion. Res2Net captures multi-scale information by processing features step by step. However, this approach can result in the loss of fine-grained information during the sequential feature transfer. In contrast, the parallel Res2Net, shown in Fig. 4, performs simultaneous convolutional operations at each scale, leading to more efficient multi-scale feature fusion. This parallel processing preserves the independence and complementarity of different scale features, further enhancing the model’s feature representation.

The parallel Res2Net module achieves parallel extraction of multi-scale features by splitting the input features into two parts $(W_1, W_2)$, urther subdividing each part into multiple groups, and applying a sequential convolution process to each group of features. First, the input features are split into two parts, and the features in each part are further divided into multiple groups $(X_1, X_2, X_3, X_4\; \text {and} \;X'_1, X'_2, X'_3, X'_4)$, with each group of features passed through successive layers of 3$\times$3 convolutions to generate new feature maps $(y_1, y_2, y_3, y_4\; \text {and} \;y'_1, y'_2, y'_3, y'_4)$. During processing, the input for each convolution layer is derived not only from the output of the preceding layer in the same group but is also connected to the residuals of features from other groups, enhancing the feature fusion between different groups. Once the convolution operation is complete, the features from the two parts are concatenated individually and finally integrated using 1$\times$1 convolution to produce the enhanced multi-scale features.

Moreover, the parallel structure mitigates the issue of information attenuation during feature transfer by processing features of different scales concurrently within the same hierarchy, without depending on layer-by-layer transfer. This design avoids the risk of gradual decay or information loss that can occur after multiple transformations in traditional stepwise structures. By maintaining both global and local information, the parallel Res2Net ensures that neither local details nor global features are weakened during the fusion process, optimizing the integrity of the feature representation.

#### ECA_block with tandem structure

**Fig. 5:**
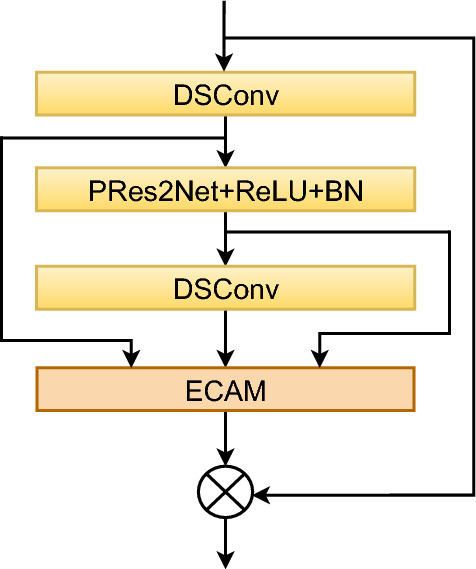
ECA_block with tandem structure.

As shown in Fig. 5, the connection of the three SE_blocks in ECAPA-TDNN is adopted in the ECA_block module, which achieves more efficient feature fusion through direct jump connections. Compared to traditional structures, this design minimizes interference from intermediate layers, allowing global information to be transferred more directly to the ECAM and preserving the integrity of the input features. This reduces information attenuation. The design using three blocks enables a deeper fusion of features, capturing low-level, mid-level, and high-level representations, which significantly enhances the model’s ability to handle complex tasks. By leveraging direct skip connections between blocks, the model ensures efficient information flow, reducing both attenuation and interference, while preserving the integrity of the features. Furthermore, this design allows for a more effective combination of global and local features, boosting the model’s selectivity and expressiveness. The use of multiple blocks also improves the model’s resilience to interference, contributing to greater stability and faster convergence during training.

The model’s input is denoted as $X_0$, with each block producing an output $X_i$. Each block operates at a different level, capturing features at varying granularities. In the first block, features are extracted to produce the output $X_1 = f_1(X_0)$. Similarly, in the second block, the output is given by $X_2 = f_2(X_1)$, and the process continues sequentially. The equation is defined as:

2 $$\begin{aligned} X_i = f_i(X_{i-1}), \quad i = 1, 2, \ldots , n \end{aligned}$$

where $f_i$ denotes the feature extraction function of the *i*-th block, and the output $X_i$ captures features across multiple levels. The use of multiple blocks enhances the model’s capacity to represent features at various scales, thereby improving its overall expressiveness, particularly in tackling complex tasks.

Skip connections address the issue of vanishing or exploding gradients by transmitting information across layers, while also improving the stability of model training. In the design of multiple regions, the output of each layer not only relies on the features of the previous layer but also directly benefits from the information passed through skip connections. The features transmitted in each layer are denoted as $X_i$, and the skip connection adds the input of the *i*-th layer, $X_0$, directly to the output of subsequent layers, resulting in the final output:

3 $$\begin{aligned} Y = X_0 + f(X_0) \end{aligned}$$

This design ensures a smoother flow of information, reducing information loss and enhancing the model’s overall expressive capacity.

### State selection space

The Selective State Space (SSS) module significantly enhances the temporal modelling capabilities in speaker verification and speech tasks by integrating a state space model (SSM), 1D convolution, and a selective feature extraction mechanism. Initially, the SSS module updates states using 1D convolution and Gated Recurrent Unit (GRU) networks, effectively capturing both long and short-temporal dependencies. This process is followed by the generation of observational features through a convolutional layer, ensuring that both local and global information are effectively captured. Furthermore, the SSS module employs a selective feature extraction mechanism that generates selective weights via an attention mechanism combining global average pooling and maximum pooling. This mechanism dynamically adjusts attention to important features while suppressing irrelevant information. The combination of 1D convolution with GRU enhances the efficiency and accuracy of modelling temporal dependencies by effectively capturing local patterns. Additionally, the dynamically generated hidden states provide the SSS module with greater adaptability in managing various sequence lengths and feature distributions.

**Fig. 6:**
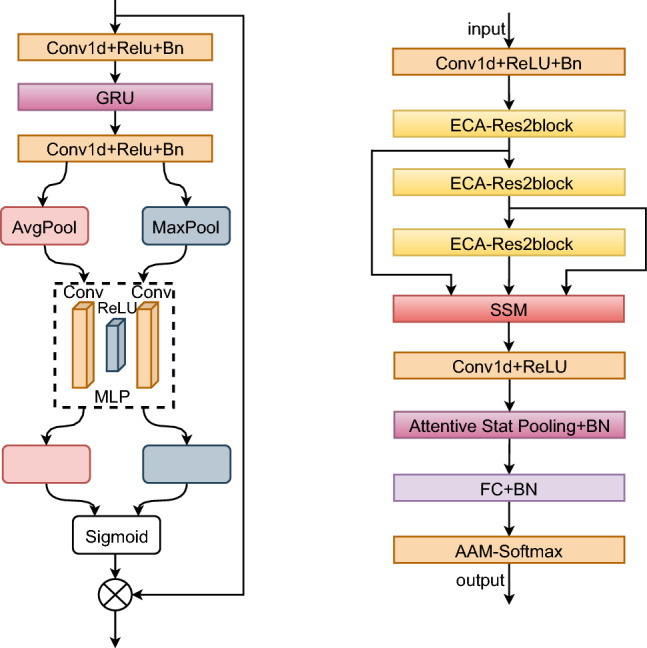
The left figure shows the structure of the SSS module and the right figure shows the structure of EPCNet with the SSS module.

The structure of the SSS module is illustrated on the left side of Figure 6. In SSS, the model first captures the temporal dynamics of the input data. Specifically, the input features are first processed through a convolutional layer, followed by batch normalization and a ReLU activation function. Long-term dependencies are then captured by a GRU network, which generates new temporal states by updating the hidden states recursively, combining the current features with past hidden states. Next, these temporal states are processed through another convolutional layer to extract salient features that can be used for subsequent tasks, which corresponds to obtaining key observations from the temporal states. Finally, the channel attention mechanism further amplifies these features and generates attention weights. These attention weights are applied to the observed features to focus on amplifying key channel information while suppressing irrelevant channel interference.

As shown on the right side of Figure 6, the SSS module is executed following the ECA_block module, which offers significant advantages by enhancing channel features before timing modelling. The ECA_block module first optimizes feature selectivity, prioritizing critical channel features while suppressing irrelevant ones. Subsequently, the SSS module conducts dynamic timing modelling on these optimized features, capturing both long and short-time dependencies to ensure accurate representation of timing features. This sequential design not only avoids information redundancy but also ensures that the SSS module processes high-quality features, thereby enhancing the model’s selectivity for timing features.

### Experimental settings

#### Experimental environment

The experiments were conducted on a Windows 10 system in the following environment: Python version 3.11.2, PyTorch version 2.0.1, and CUDA version 12.4. All networks were trained on an NVIDIA GeForce RTX 3090 GPU (24 GB). The specific hyperparameter settings are shown in Table 1.

**Table 1:** Training setup of the network.

Hyperparameter	Value
Epochs	60
Optimizer	Adam
Learning rate	0.001
Weight decay	$1*10^{-5}$
Batch size (training)	128
Batch size (eval)	128
Frequency mask width	[0, 8]
Time mask width	[0, 10]

#### Dataset

The proposed model is evaluated using the CN-Celeb1 dataset, one of the most adopted open-source datasets.CN-Celeb1 contains more than 130,000 discourses from 1,000 Chinese celebrities, covering 11 different genres in the real world. The evaluation set is the CnCeleb-test (Cn-test) covering 197 speakers and 17777 discourses. To ensure the generalisation of the experiments, we also use the Voxceleb1 dataset to evaluate the model efficiently, where Voxceleb1 contains more than 100,000 speech segments from 1,251 celebrities, covering a wide range of celebrities and speech scenarios, ensuring the generalisation ability of the model. The evaluation set is vox_test and contains 158 speakers.

#### Preprocessing

To ensure that only speech samples of valid length are used in the training process, the minimum and maximum durations of the audio data are set to 0.5 seconds and 3 seconds, respectively. The audio sampling rate is set to 16 kHz, allowing for a comprehensive representation of the spectral information in the speech signal. Additionally, all audio samples are volume normalized to a target level of -20 dB, minimizing volume discrepancies that may arise from varying recording environments or equipment.

In the preprocessing stage, we employed a Filter Bank (Fbank) as the feature extraction method, extracting 80-dimensional Mel filter bank features. To enhance the model’s robustness, several data augmentation techniques were applied during the experiments. Speed perturbation was utilized to create three types of samples with different speech speeds: slow, normal, and fast, thus increasing data diversity. To further improve the model’s adaptability to noise, noise enhancement was applied with a probability of 20%. Additionally, we employed the SpecAug data augmentation method to mitigate overfitting by randomly masking certain frequencies and time periods. Specifically, the frequency mask width ranges from 0 to 8 frequency bands, while the time mask width ranges from 0 to 10 time bands.

#### Loss function

AAMLoss (Additive Angular Margin Loss)^37^ was used as the loss function in the experiments to improve speaker differentiation by increasing the inter-class angular spacing. The initial value of the angular spacing is 0.2 and is gradually adjusted to 0.3. The scaling factor is 32. To further enhance the effectiveness of the model training, the margin scheduler is enabled to dynamically adjust the margin parameter in the loss function as the training progresses. The formula is given by the following equation:

4 $$\begin{aligned} L = -\frac{1}{N} \sum _{i=1}^{N} \log \frac{e^{s \cdot (\cos (\theta _{y_i} + m))}}{e^{s \cdot (\cos (\theta _{y_i} + m))} + \sum _{j \ne y_i} e^{s \cdot \cos \theta _j}} \end{aligned}$$

where $\theta _{y_i}$ is the angle of the input sample with respect to the correct category weights. *m* is the angular spacing (margin), used to increase the angular distance between classes and enhance category differentiation. *s* is the scaling factor, used to adjust the length of the angular vectors. $y_i$ is the true category label of the input sample, and *n* is the batch size.

#### Training protocols and assessment indicators

The model is trained using the Adam^38^ optimizer with an initial learning rate of 0.001^9^, in conjunction with the WarmupCosineSchedulerLR scheduler. To prevent the model from converging too quickly in the initial stages and to ensure stability during training, the learning rate is gradually increased from $1 \times 10^{-5}$ to 0.001 over the first 5 epochs. Following this period, the learning rate is gradually reduced to a minimum of $1 \times 10^{-5}$. The model is trained for a total of 60 epochs with a batch size of 128. During the evaluation phase, the batch size remains at 128, while the maximum duration of the evaluation audio is limited to 20 seconds.

We test the model at the final epoch and report all system performances based on Equal Error Rate (EER), Minimum Detection Cost Function (minDCF), and Accuracy (ACC). EER indicates the point at which the rates of acceptance and rejection errors are equal. MinDCF incorporates the weights of acceptance and rejection errors, denoted as follows:

5 $$\begin{aligned} C_{\text {def}} = C_{\text {miss}} \times P_{\text {miss}} \times P_{\text {tar}} + C_{\text {fa}} \times P_{\text {fa}} \times (1 - P_{\text {tar}}) \end{aligned}$$

where $$p_{\text {target}} = 0.01$$, $$C_{\text {fa}} = C_{\text {miss}} = 1$$.

### Experimental results

In this section, we perform a series of comparison experiments using the Cn-Celeb1 dataset to assess the feasibility of the proposed approach. To evaluate the effectiveness of the EPCNet structure, we first compare it against several mainstream models for voiceprint recognition, as presented in Table 2.

**Table 2:** Model comparison.

Model	EER	minDCF	ACC(%)	Params (M)
TDNN(C=512)^39^	0.185	0.812	84.4	3.2
ECAPA-TDNN(C=512)^9^	0.179	0.799	89.1	6.9
Res2Net^40^	0.218	0.784	90.1	5.6
EResNet^41^	**0.148**	**0.685**	94.5	6.6
ResNetSE^42^	0.157	0.732	93.6	7.8
EPCNet-TDNN(C=512)	0.154	0.724	**95.7**	13.9

As shown in the table of experimental results, the performance of different models on the speech recognition task varies significantly. The EPCNet-TDNN (C=512) model achieves the highest accuracy, reaching 95.7%, with an Equal Error Rate (EER) of 0.154 and a minimum Detection Cost Function (minDCF) of 0.724, demonstrating a well-balanced performance that is suitable for scenarios requiring high accuracy. In contrast, the TDNN (C=512) and ECAPA-TDNN (C=512) models perform relatively poorly, particularly the former, with an EER of 0.185 and an accuracy of only 84.4%. Although the Res2Net model achieves an accuracy of 90.1%, its EER of 0.218 and high error rate negatively affect its overall performance. The ResNetSE model is comparable to EResNet, with an EER of 0.157, a minDCF of 0.732, and an accuracy of 93.6%, but it remains weak in error rate control. While EResNet excels in EER and minDCF, there is still room for improvement in accuracy compared to EPCNet. The EPCNet-TDNN model is enhanced in terms of feature extraction, temporal modelling and channel selectivity by introducing a number of improvements. By combining ECA_Block with Selective State Space Model (SSS), the proposed model captures both local and global features of speech signals using residual networks, state space modelling and attention mechanisms.Overall, despite its large number of parameters, it is worthwhile in terms of performance improvement. The EPCNet-TDNN (C=512) model is particularly noteworthy as it excels in several metrics.

In addition to this the actual results of TDNN, ECAPA-TDNN and our proposed EPCNet-TDNN are compared with the number of channels of 128, 256 and 512, as shown in Tables 3, 4, 5.

**Table 3:** Number of channels is 128.

Model	EER	MinDCF	ACC(%)	Params (M)
TDNN	0.240	0.899	70.6	0.83
ECAPA-TDNN	0.208	0.851	76.3	1.3
EPCNet-TDNN	0.194	0.773	82.9	2.3

**Table 4:** Number of channels is 256.

Model	EER	MinDCF	ACC(%)	Params (M)
TDNN	0.255	0.824	77.4	1.3
ECAPA-TDNN	0.209	0.793	85.3	2.5
EPCNet-TDNN	0.192	0.775	94.1	5.8

**Table 5:** Number of channels is 512.

Model	EER	MinDCF	ACC(%)	Params (M)
TDNN	0.185	0.812	84.4	3.2
ECAPA-TDNN	0.179	0.799	89.1	6.9
EPCNet-TDNN	0.154	0.724	95.7	13.9

As shown in the table, increasing the number of channels from 128 to 512 results in varying degrees of change across the metrics—EER, minDCF, and accuracy—for TDNN, ECAPA-TDNN, and the proposed model. When the number of channels is set to 128, the ECPNet-TDNN achieves the best performance, with an EER of 0.194, a minDCF of 0.773, and an accuracy of 82.9%, which is considerably better than the results of TDNN and ECAPA-TDNN. This suggests that EPCNet-TDNN demonstrates strong feature extraction capabilities even at a lower number of channels. As the number of channels increases to 256, TDNN’s EER rises, while ECAPA-TDNN and the proposed model improve. Notably, the EER of the proposed model decreases to 0.192, and its accuracy significantly increases to 94.1%, demonstrating better robustness. When the channel count is further increased to 512, all models show a significant reduction in EER and minDCF, along with substantial improvements in accuracy. In particular, the proposed model exhibits an EER of 0.154, a minDCF of 0.724, and an accuracy of 95.7%, excelling across all metrics. Overall, the proposed model demonstrates clear performance advantages at different channel counts, especially at 512 channels, confirming its effectiveness in terms of EER, minDCF, and accuracy.

To further validate the effectiveness of the proposed enhancement strategy, we conduct an ablation experiment to investigate the impact of different strategies on object detection performance. Using the same test conditions, we adopt ECAPA-TDNN(512) as the baseline model and incrementally integrate the proposed enhancement strategies. Additionally, during our exploration of the ECA_block module, the structure was found to be similar to the three closely linked SE-Res2block modules in ECAPA-TDNN referring to it as a tandem structure (TS). To determine the effectiveness of this structure within the ECA_block, we conclude the ablation experiments by comparing the model’s performance with and without its inclusion. The experimental results are presented in Table 6.

**Table 6:** Ablation experiment.

ECAM	DSConv	PRS	SSS	TS	EER	minDCF	ACC(%)	Params(M)
					0.179	0.799	89.1	6.9
$\checkmark$					0.168	0.752	91.9	6.2
	$\checkmark$				0.178	0.797	89.8	6.7
		$\checkmark$			0.173	0.791	90.2	6.8
			$\checkmark$		0.167	0.750	92.5	14.9
$\checkmark$			$\checkmark$		0.160	0.741	94.1	14.2
	$\checkmark$	$\checkmark$			0.171	0.783	91.0	6.5
$\checkmark$		$\checkmark$	$\checkmark$		0.158	0.731	94.7	14.1
$\checkmark$	$\checkmark$	$\checkmark$	$\checkmark$		0.156	0.728	95.1	-
$\checkmark$	$\checkmark$	$\checkmark$	$\checkmark$	$\checkmark$	0.154	0.724	95.7	13.9

In this ablation experiment, we analyze the impact of individual modules on model performance by systematically removing or combining different components (ECAM, DSConv, PRS, and SSS) within the ECAPA-TDNN framework. The experimental results indicate that the combination of these modules significantly influences the model’s accuracy and robustness. Notably, including ECAM and SSS modules yields the most substantial improvements, particularly by enhancing accuracy and reducing the Equal Error Rate (EER), underscoring their effectiveness in performance optimization. The synergy between these two modules is especially crucial for enhancing the model’s discriminative ability and stability. In comparison, the PRS and DSConv modules exert a more limited impact, with their minimal contributions to performance suggesting a relatively weak role in feature extraction. In addition, we found that when the CRB structure is introduced into the model, it has an effect on the model. When all modules are integrated, the model achieves optimal performance, indicating that the modules complement each other in feature extraction and information gain. Overall, the results demonstrate that the ECAM and SSS modules are pivotal to performance improvement, while the contribution of DSConv remains modest, with the fully integrated model achieving the best results.

In addition, to further validate the robustness of the model, we further validate the performance of our method in the speaker verification (SV) task on the Voxceleb1 dataset, and similarly, we compare it with several mainstream voiceprint recognition models in order to evaluate the effectiveness of the EPCNet structure, as shown in Table 7.

**Table 7:** Experimental results in the Voxceleb1 development dataset.

Model	EER	minDCF	ACC (%)	Params (M)
TDNN(C=512)^39^	0.112	0.806	94.6	3.2
ECAPA-TDNN(C=512)^9^	0.0654	0.514	98.5	6.9
Res2Net^40^	0.104	0.779	96.1	5.6
EResNet^41^	0.0753	0.651	97.2	6.6
ResNetSE^42^	0.0896	0.715	97.6	7.8
EPCNet-TDNN(C=512)	0.0576	0.423	98.2	13.9

Table 7 shows the results of our experiments on the Voxceleb1 development dataset, comparing the EER, minDCF, ACC, and the number of parameters (Params) of the different models, respectively. It can be seen that our method (EPCNet-TDNN) performs on several performance metrics, further validating its effectiveness on the Voxceleb1 dataset.

## Conclusions

This paper proposes an enhanced speaker verification architecture, EPCNet-TDNN, which systematically addresses the limitations of existing TDNN base models through a series of innovative interrelated improvements. The introduction of the ECAM mechanism effectively combines channel and spatial attention, achieving superior feature selectivity, while the parallel residual structure enables multi-scale feature extraction to be performed independently. The serial structure design promotes efficient information flow, while the selective state space module enhances temporal modeling capabilities by combining the state space model with the selective attention mechanism.

Comprehensive experimental validation on the CN-Celeb1 and VoxCeleb1 datasets demonstrates that the model achieves performance improvements across all metrics and maintains consistent performance improvements across different channel configurations. Ablation studies confirm that the ECAM and SSS modules are the primary contributors to performance improvements, and their synergistic integration achieves optimal results. These improvements enable more reliable speaker verification in challenging real-world scenarios, particularly with short audio clips and variable acoustic conditions.

The proposed architecture achieves significant progress in speaker verification. By decoupling feature extraction through parallel processing, EPCNet-TDNN reduces the inherent information loss in sequence-based architectures while providing a comprehensive attention mechanism that surpasses traditional channel-based methods. However, the increased computational complexity necessitates further research on model compression and efficiency optimization in the future. Future work will focus on validating generalization capabilities on large-scale, diverse datasets and optimizing the adaptive kernel selection mechanism and PRS group configuration to enhance performance across different speech features.

## Data Availability

The dataset used in this paper is from the publicly available dataset at: https://openslr.org/82/
